# 4D Printing: The Development of Responsive Materials Using 3D-Printing Technology

**DOI:** 10.3390/pharmaceutics15122743

**Published:** 2023-12-07

**Authors:** Pablo Edmundo Antezana, Sofia Municoy, Gabriel Ostapchuk, Paolo Nicolás Catalano, John G. Hardy, Pablo Andrés Evelson, Gorka Orive, Martin Federico Desimone

**Affiliations:** 1 Universidad de Buenos Aires, Consejo Nacional de Investigaciones Científicas y Técnicas (CONICET), Instituto de la Química y Metabolismo del Fármaco (IQUIMEFA), Facultad de Farmacia y Bioquímica Junín 956, Piso 3, Buenos Aires 1113, Argentina; pablo.e.antezana@gmail.com (P.E.A.); smunicoy@gmail.com (S.M.); 2 Universidad de Buenos Aires, Consejo Nacional de Investigaciones Científicas y Técnicas (CONICET), Instituto de Bioquímica y Medicina Molecular (IBIMOL), Facultad de Farmacia y Bioquímica, Buenos Aires 1428, Argentina; pevelson@ffyb.uba.ar; 3 Instituto de Nanociencia y Nanotecnología (CNEA-CONICET), Nodo Constituyentes, Av. Gral. Paz 1499 (B1650KNA), San Martín, Buenos Aires 8400, Argentina; ostapchukgabriel@gmail.com (G.O.); paoloncatalano@gmail.com (P.N.C.); 4 Departamento de Micro y Nanotecnología, Gerencia de Desarrollo Tecnológico y Proyectos Especiales, Gerencia de Área de Investigación, Desarrollo e Innovación, Centro Atómico Constituyentes, Comisión Nacional de Energía Atómica, Av. Gral. Paz 1499 (B1650KNA), San Martín, Buenos Aires 8400, Argentina; 5 Universidad de Buenos Aires, Facultad de Farmacia y Bioquímica, Departamento de Ciencias Químicas, Cátedra de Química Analítica Instrumental, Junín 954, Buenos Aires 1113, Argentina; 6 Materials Science Institute, Lancaster University, Lancaster LA1 4YB, UK; j.g.hardy@lancaster.ac.uk; 7 Department of Chemistry, Faraday Building, Lancaster University, Lancaster LA1 4YB, UK; 8 NanoBioCel Research Group, School of Pharmacy, University of the Basque Country (UPV/EHU), 01006 Vitoria-Gasteiz, Spain; gorka.orive@ehu.es; 9 Bioaraba, NanoBioCel Research Group, 01009 Vitoria-Gasteiz, Spain; 10 Biomedical Research Networking Centre in Bioengineering, Biomaterials and Nanomedicine (CIBER-BBN), Institute of Health Carlos III, Av Monforte de Lemos 3-5, 28029 Madrid, Spain; 11 University Institute for Regenerative Medicine and Oral Implantology—UIRMI (UPV/EHU-Fundación Eduardo Anitua), 01007 Vitoria-Gasteiz, Spain

**Keywords:** 3D printing, stimuli responsive materials, 4D printing, drug delivery, smart materials

## Abstract

Additive manufacturing, widely known as 3D printing, has revolutionized the production of biomaterials. While conventional 3D-printed structures are perceived as static, 4D printing introduces the ability to fabricate materials capable of self-transforming their configuration or function over time in response to external stimuli such as temperature, light, or electric field. This transformative technology has garnered significant attention in the field of biomedical engineering due to its potential to address limitations associated with traditional therapies. Here, we delve into an in-depth review of 4D-printing systems, exploring their diverse biomedical applications and meticulously evaluating their advantages and disadvantages. We emphasize the novelty of this review paper by highlighting the latest advancements and emerging trends in 4D-printing technology, particularly in the context of biomedical applications.

## 1. Introduction

Tissue engineering has been widely revolutionized with the development of 3D-printed structures where the precise fabrication of materials with functional structures can be produced in a mass, batch, or indeed patient-specific fashion. These 3D-printed structures can be composed of bioinert materials (e.g., metals/alloys) designed to stay in place for a long period of time (e.g., orthopedics); or indeed degradable materials (e.g., natural/synthetic polymers) which may eventually be replaced by the patient’s tissues over time. In both cases, their lifetimes will be dictated by the stresses that they are exposed to (e.g., wear and tear on load-bearing materials like acetabular cups).

There are different types of additive manufacturing (AM) that may be used to produce responsive materials [1,2,3]. Such processes include: powder bed fusion (regions of powder (commonly metal) are selectively melted using a laser, another layer of powder is deposited, and this process is performed repeatedly) [4,5]; binder jetting (liquid binder is deposited/jetted over a region of powder, additional powder is deposited, and this is repeated) [6]; directed energy deposition (laser melting of metals extruded from nozzles) [7]; material extrusion (molten thermoplastics are extruded from a nozzle) [8]; material jetting (regions of layers of liquid resin are cured (often with light), another layer of liquid resin is deposited, and this is repeated) [9]; stereolithography (regions of layers of liquid resin deposited on a print bed are UV-cured, the print bed is lowered, another layer of liquid resin is deposited, and this is repeated) [10]; sheet lamination (sheets of materials are cut to shape, another sheet deposited and made to adhere and then cut to shape, and this is repeated) [11]; electrospinning to deposit fibers onto a substrate to create a desired pattern (offering high print resolution and control over fiber orientation) [12,13], extrusion bioprinting (bioink composed of a mixture of cells and other materials are extruded from a nozzle followed by crosslinking or curing); inkjet bioprinting (droplets of bioink composed of a mixture of cells and other materials are jetted onto a print bed) [14,15]; laser-assisted bioprinting (laser pulses are used to generate droplets of bioink composed of a mixture of cells and other materials dropped onto a surface); multiphoton fabrication (an ultrafast laser is used to print structures inside other structures, including living organisms) [16]. The choice of which technique will be employed depends on the choice of materials used and the structure to be printed.

**Figure 1:**
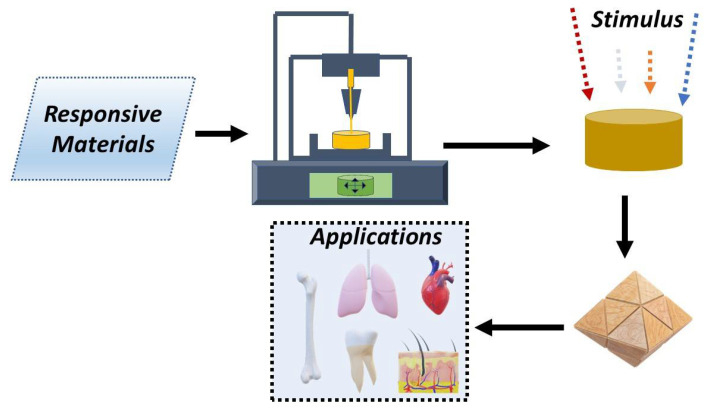
Schematic illustration of the 4D-bioprinting process.

Although 4D printing employs the same printing techniques as 3D printing, the materials employed are stimuli-responsive, which means that the structures printed can change their shape and/or function over time in response to external stimuli (e.g., temperature, light, electric fields, and magnetic fields) [17,18] (Figure 1).

The use of 4D printing has gained popularity in biomedicine because of its potential to facilitate disease modeling and drug testing [19] via the creation of anatomically correct organoid models, and novel tissue engineering paradigms (wherein the creation of complex functional structures that can recreate the properties of natural tissues can help the tissues to regenerate) [20,21,22,23,24]. In this review, we discuss recent advances in 4D bioprinting for biomedicine including the different materials used for 4D bioprinting, the external stimuli available, and the possible applications of this technology. This work can serve as a starting point for deeper research on 4D printing and its implications in various fields and offers the opportunity to explore and better understand the key aspects of this emerging technology.

## 2. Advantages and Disadvantages of Different 3D-Printing Techniques

### 2.1. Selective Laser Sintering (SLS)

Power bed fusion and binder jetting are both techniques that use the selective laser sintering method. The primary advantage of the SLS method lies in its exceptional fracture toughness and mechanical strength, ensuring high-quality outcomes. Additionally, this technique offers the notable benefit of creating components without the need for supporting structures [25]. Consequently, each build can yield more parts, reducing the post-processing requirements. However, it is essential to note that the strength of parts may vary, introducing the potential for different strength levels among multiple copies of the same part. In addition, there is a wide range of available biomaterials to use in this method. Compared to conventional techniques, the controlled pore size of the scaffold in SLS contributes to enhanced tissue regeneration. Nevertheless, the high temperature generated during the laser radiation makes it unsuitable for cell printing [26,27,28,29].

### 2.2. Directed Energy Deposition (DED)

Producing porous implants using DED offers advantages over conventional methods. The ability to modify mechanical properties by altering the orientation or geometry of the build is a notable advantage. Additionally, DED allows for the incorporation of different materials, achieving optimal properties through functionally graded materials. The process is also more adaptable to customizing implants based on specific patient requirements, making it easier to tailor the implants to individual needs [30]. Using DED for cladding two dissimilar materials offers an additional advantage, thanks to the potential utilization of functionally graded alloys. Another valuable cladding technique with DED is multi-axis cladding, enabling the deposition of layers at any angular axis. This functionality stands as a significant advantage of DED over other additive manufacturing systems [31,32,33]. On the other hand, important disadvantages of DED processes include low repeatability, poor adaptive control, accumulative error, and the need for expensive post-processing technologies [34].

### 2.3. Material Extrusion

It is crucial to underscore both the advantages and disadvantages of the material extrusion technique. One notable benefit is its simplicity and safety, as it does not involve toxic materials and is user-friendly. Moreover, post-printing, the only additional step required is support removal, making it easily manageable. Customization is another strength, allowing users to produce solid or porous parts by adjusting parameters such as the fill pattern, raster width and angle, and air gaps using the software. In the context of tissue engineering, the simplicity of this method stands out [35,36]. However, there are some drawbacks to consider. Accuracy may be compromised, leading to a grainy surface due to the layer-by-layer deposition process through the nozzle. The slow printing speed, a consequence of using a single nozzle tip for layering, can also extend the time required to complete a part. Additionally, each new material must adhere to the diameter requirements of the nozzle tip in the printer. In the context of biomaterial printing, limitations arise from the high temperature used in the process, preventing cell printing and requiring a separate step for cell seeding. Resolution is another concern, being lower compared to alternative methods. Lastly, materials may lead to nozzle clogging, presenting an additional challenge [37,38].

### 2.4. Material Jetting

The adaptability of technology to accommodate thin layer thicknesses allows for the production of high-quality parts, reducing the visibility of staircase effects and enhancing the printing of thin wall features. Another advantage is the low surface roughness texture, addressing a significant challenge in many additive manufacturing technologies. Additionally, material jetting technology eliminates the need for post-processes, as parts are usable in their as-built condition after separation from the build platform and support removal processes [39,40]. On the other hand, some disadvantages are the high cost, the slow printing process, high dimensional accuracy, and poor mechanical properties [41].

### 2.5. Stereolithography (SLA)

SLA printing displays a wide range of advantages, including a stable printing process and the highest resolution when compared to other printing techniques. This superior resolution stands in contrast to the ones found in other printers. Another noteworthy advantage is the capability to produce large-size models. However, it is important to note that the printing rate is inversely proportional to the size of the models, as larger models result in slower printing rates due to the laser beam movement dependency [42]. Despite these strengths, the SLA printing process is characterized by its inherent slowness, attributed to the low photopolymerization rates during printing. The process is “discontinuous” because it involves separate steps, including laser scanning, platform movement, and resin refill, creating intervals with no printing process. An additional drawback is the limitation on certain biocompatible resins that cannot be used in this printing system, and the inability to print cells. This limitation arises from the potential damage to DNA and promotion of cell lysis caused by the UV irradiation used in the SLA process [43].

### 2.6. Sheet Lamination

Certainly, there are several advantages associated with the sheet lamination method. It can be employed for various materials, including polymers, paper, ceramics, and metals, with a suitable method used to bind the sheets of each material. Additionally, this process is known for being cost-effective, exhibiting a high printing speed, and offering ease of material handling. The primary disadvantage of the sheet lamination process is its limitation in printing complex geometries. Furthermore, this method tends to produce outcomes with a resolution ranging from poor to average [41].

### 2.7. Electrospinning

The primary advantage of this method lies in its versatility, since it can be employed to synthesize fibers derived from a combination of polymers, enhancing their characteristics. Additionally, it enables the creation of scaffolds with a high surface area and a structure similar to the extracellular matrix, promoting favorable cell interaction and growth. However, a notable disadvantage is that cell migration is occasionally impeded due to the small pores formed between the microfibers and nanofibers obtained [44].

### 2.8. Extrusion Bioprinting

A significant advantage of this method is its scalability, facilitated by a continuous bioink flow and a large deposition rate. Furthermore, it accommodates high viscosity bioinks and high cell concentrations. However, a drawback of this technique is the requirement for bioinks with shear-thinning properties. In addition, it exhibits a lower resolution compared to other methods and nozzle clogging represents another disadvantage [36].

### 2.9. Inkjet Bioprinting

The advantages of inkjet-based bioprinting encompass high print speeds, cost-effectiveness, high resolution, and wide availability. However, challenges include low droplet directionality and unreliable cell encapsulation, primarily attributed to the low concentration of the ink [45].

### 2.10. Laser-Assisted Bioprinting

An interesting advantage of this printing technique is its non-contact process, effectively eliminating nozzle clogging. Furthermore, it has a high resolution, allowing for the printing of single cells per droplet, utilization of high-cell densities, and handling of low-viscosity cell suspensions. However, there are notable disadvantages to consider, with the most significant being the risk of photonic cell damage arising from laser exposure. Additionally, the use of metals as a laser-energy-absorbing layer raises concerns about the cytotoxicity induced by metallic nanoparticles. Furthermore, scalability is limited due to the elevated cost of the laser system and the complexity associated with controlling laser pulses [46,47,48].

### 2.11. Multiphoton Fabrication

The main advantage of the multiphoton fabrication is the creation of high-performance, 3D photonic crystals and microinductors. Furthermore, there is potential for commercial applications in other domains, including tissue scaffolding and the development of functional 3D components for microfluidic systems [49]. In addition, this method has the ability to fabricate computer-designed, fully 3D structures with a resolution surpassing the diffraction limit. This method uses light to drive the reaction instead of heat. This implies several advantages, including the elimination of solvent, high reaction rates at room temperature, and spatial control of polymerization. However, a notable disadvantage is the limitation on the number of designs that can be structured. In many cases, the materials have not been able to deliver the necessary resolution and structural integrity required for optical metamaterials [50].

## 3. Materials

In this section, we will discuss the different materials used for 4D printing, which are usually named “smart materials” that are employed in 4D printing due to their ability to change their properties over time in response to stimuli [51].

### 3.1. Shape Memory Materials (SMM)

SMMs are also known as intelligent materials capable of altering and adapting their shape in response to external stimuli. These materials include shape memory polymers (SMPs), shape memory alloys (SMAs), shape memory ceramics (SMCrs), and others. The SMM materials are in a metastable state and are able to switch from a temporary state to a stable state [52] due to the exposure to changes in pH, moisture, and electrical and magnetic fields [53,54,55,56].

#### 3.1.1. Shape Memory Polymers (SMPs)

SMPs are a type of smart, stimuli-responsive material capable of changing their shape, in a controlled manner, when an external stimulus is applied [57,58]. These materials respond to different stimuli (e.g., temperature, pH, light [59], etc.) and their shape changes can be reversible [60,61]. SMPs respond to temperature transitions such as the glass transition temperature (Tg) and melting temperature (Tm) through physical and chemical cross-links. Typically, these materials are processed and molded at temperatures above their Tg to establish their initial shape. Upon cooling below the Tg, the shape becomes deformed and remains fixed in that position [62]. SMPs display several advantages over other materials, such as a more reliable recovery performance, lighter weight, biodegradability, and low toxicity [63,64]. Thermo-responsive SMPs may include blocks composed of polycaprolactone (PCL), poly(lactic acid) (PLA), acrylated epoxidized soybean oil (AESO), and polyurethane (PU) [65,66,67]. For example, the U.S. Food and Drug Administration compliant biodegradable polyester PLA is popular for inclusion in SMP materials owing to its low Tg [68], and the ease with which it is possible to impart other properties via the preparation of composites that respond to stimuli including temperature [69], light [70], and magnetic fields [71].

#### 3.1.2. Shape Memory Alloys (SMAs)

SMAs are a metal alloys that go through a solid-to-solid phase transformation [72]. There are usually two phases of the process that could depend on changes in temperature or magnetic field [73].

In the case of temperature, the shape memory effect arises from the presence of two crystal structures within the alloys: the martensite phase at low temperatures and the austenite phase at high temperatures. SMAs are deformed in the martensite phase and regain their original shape as the temperature increases and transitions to the austenite phase [74]. In the case of magnetic fields, there are two approaches used: magnetically-induced reorientation and magnetic-field-induced phase transformation [75].

In general, the use of SMAs needs the addition of Ni-Ti, Cu-Al-Ni, or Fe-Mn-Al-Ni, among others [76,77,78,79]. In this vein, the most frequently used printing techniques are selective laser sintering (SLS) and selective laser melting (SLM) due to their capacity for printing metallic structures with adequate strength characteristics.

When comparing SMP with SMA, SMP materials are preferred since they can achieve a higher shape recovery, the stiffness could be tailored to their Tg range, they have lower cost, high elastic deformation, and can be designed to be biodegradable and biocompatible, making them useful for biomedical applications [63,80,81].

#### 3.1.3. Shape Memory Ceramics (SMCrs)

SMCrs, like SMAs, exhibit either superelasticity, allowing them to deform significantly and recover, or the shape memory effect, enabling transformation between predefined states with the assistance of an external stimulus. Certain brittle ceramics also undergo martensitic transformations and can exhibit shape memory effects akin to SMAs, but their brittle nature poses concerns regarding cracking [82]. Direct ink writing, SLS, and SLM are the printing techniques that are used to print ceramics inks [83,84].

#### 3.1.4. Other Materials

In addition to the commonly used SMPs, SMAs, and shape memory ceramic-reinforced composites (SMCrs), there are also shape memory hybrids and composites (SMHs, SMCs). Shape memory composites are multiphase materials that blend a filler and matrix phase on a larger scale. Conversely, SMHs involve the combination of two materials at the molecular or nanometer level. The transformations displayed by SMHs and SMCs depend on the specific material component possessing the shape memory capability. For instance, an SMA wire integrated into a polymer matrix undergoes martensitic changes, whereas an SMH consisting of different polymer phases experiences physical and chemical cross-links associated with temperature transitions [85].

### 3.2. Gels

In addition to SMPs, stimuli-responsive gels are a popular material used for 4D printing since they display a high shape-morphing capability in response to the different stimulus [86,87,88,89].

#### 3.2.1. Hydrogels

Hydrogels are cross-linked polymeric networks swollen with water. Due to the crosslinking between chains, these materials can absorb a large amount of water without dissolving. Hydrogels are potentially capable of folding, stretching, and bending, and experience geometrical expansion, leading to a high printing capability [90]. Moreover, they are easy to print with incorporated bioactive molecules [58,59] that can support cell growth and differentiation [20,91,92]. Smart hydrogels have physical or chemical properties that can be controlled when they are exposed to external stimuli [93,94]. A wide variety of polymers have been used in hydrogels for 4D bioprinting, including: poly(ethylene glycol) (PEG), poly(N-isopropylacrylamide) (PNIPAM), poly-N-vinyl caprolactam (PNVCL), collagen, gelatin, and alginate [95].

The most common polymer used in hydrogels for 4D bioprinting is the U.S. FDA compliant PEG due to its biocompatibility [96,97]. PEGs can be engineered to be responsive to light [98], pH [99], moisture, and temperature [100]. PNIPAM-based hydrogels which can be engineered to be light-responsive [101] and thermo-responsive in a reversible manner were made using 4D-bioprinting technology [102]. PNVCL is water-soluble and thermally-responsive with a liquid-to-gel transition in the range of 34–37 °C [103].

Collagen is often included in bioinks because it is a natural component of the extracellular matrix [104,105,106]. The rheological properties of collagen-based hydrogels can be adapted to enable the precise printing of complex structures potentially including cells during the bioprinting process [107]. The partially hydrolyzed version of collagen, gelatin, is widely used in biomaterials [108,109,110] and undergoes a phase transition that changes its mechanical properties in response to temperature changes [19].

#### 3.2.2. Organogels

Organogels exhibit similarities to hydrogels in their ability to expand when placed in a liquid medium. These gels have a particular characteristic of swelling significantly in a specific organic solvent, which should possess comparable solubility parameters to the polymer network. Like other materials, organogels can undergo reversible changes in shape, responding to various stimuli [111,112]. Two primary printing techniques employed for organogels are digital light processing and direct ink writing [113,114].

### 3.3. Liquid Crystalline Elastomers (LCE)

LCEs are another stimulus-responsive materials with bidirectional actuation behavior that responds to several stimuli, including light, heat, magnetic fields, and electricity [115,116,117,118]. In addition, LCEs display reversible actuation to the different stimulus mediated by their molecular orientations, pitch, or mesophase-isotropic phase transition, without the need for an aqueous environment or a connected power supply. LCEs are soft materials with desirable programmable properties, characterized by a combination of the soft elasticity of lightly crosslinked polymer networks and the director-dependent, reversible property change behavior of liquid crystals [115,116,119,120,121,122,123,124,125].

There are several printing techniques that could be used with these materials, such as direct ink printing, fused deposition modeling (FDM), stereolithography, and inkjet printing [126,127,128,129,130,131]. Up to now, no biomedical devices has been approved for humans using LCE-based materials. However, there are several developments in the biomedical area such as surgical tools and cell culture set ups [132].

### 3.4. Other Functional Materials

#### 3.4.1. Magneto-Responsive Materials

Magnetic nanoparticles (MNPs) are appealing for biomedical applications because magnetic fields can penetrate all bodily tissues [133]. The most commonly used MNPs are the ferromagnetic nanoparticles, examples of which include magnetic composites such as agar/PEG-based hydrogels including Fe_3_O_4_ NPs [134], and Fe-based NPs in poly(dimethylsiloxane) (PDMS) [135].

#### 3.4.2. Electro-Responsive Materials

Electro-responsive materials may undergo deformation when exposed to electric fields, examples of which include polypyrrole (PPy) and polythiophene derivatives [136], carbon nanotubes (CNTs), graphene, and other NPs [137], often combined with other biomaterials to make bioinks with different physicochemical properties [60].

## 4. Stimulus and Effect on Responsive Materials

The new age of materials for biomedical applications involves the development of smart platforms that can respond to external stimuli and, in this way, achieve more personalized treatments and precise medicine [138]. In 4D bioprinting, materials should have good printability and shape-morphing ability, i.e., their final shape should be controlled by external triggers [139]. These can be categorized into physical, chemical, and biological stimuli. Physical stimuli include temperature, magnetic field, light, sound, and electric fields; chemical triggers comprising pH, solvent, and humidity; and biological stimuli involving enzymes, glucose, proteins, and cell traction forces, and these can be harnessed to control the morphology or functionality of the responsive material. The exposure of stimuli-responsive materials to one or more external triggers can result in irreversible/reversible deformations defining the final shape of the material. In this section, some of the different external stimuli used for 4D biomaterials are discussed.

### 4.1. Physical Stimuli

#### 4.1.1. Light

Light-responsive materials can experience a physical or chemical change by capturing the light with chromophores included in the main chain of the polymer [140]. Due to the presence of different chromophores, such as azobenzenes, spiropyrans, and fulgides, exposure to light irradiation produces changes in the chemical structure, charges, affinity for water, and polarity of the polymer that are transferred to changes in the final properties of the 4D-printed material [141]. Light-responsive biomaterials can be remotely stimulated by ultraviolet (UV), infrared (IR), and near-IR (NIR) radiation (depending on the chromophores included) where the intensity and wavelength can be precisely tuned. The main advantages of light stimuli are that they can be rapidly applied, quickly switched, and easily focused in time and space with high precision by non-/minimally-invasive techniques [142].

The type of the photochemical functional group used in the SMPs is a crucial factor in the design of light-responsive 4D materials. Based on the nature of the photosensitive moieties and the type of polymer material, the light-induced transformation can be reversible or irreversible. Among the light-responsive polymers, there are hydrogels, composites, liquid crystal elastomers, and SMPs [143]. NIR-sensitive nanocomposites that can be reversibly shape-modified by a photothermal approach [144] have been prepared in which photothermally-responsive graphene nanoplatelets were included in a temperature-responsive epoxy based on bisphenol A diglycidyl ether and the final composite was printed by fused deposition modeling. Graphene absorbs NIR radiation which results in a temperature increase that induces a change in the shape of the nanocomposites through a “thermomechanical reprogramming” process. The advantage of this photothermal method over direct thermal processes is that NIR light stimulation is more controllable. It has been shown that after exposing the nanocomposite to an NIR laser, the 4D-printed material exhibited a gradual shift of its shape in a well-defined time and position without a complex predesign.

An interesting study explored the preparation of a cholesteric liquid crystal (“azo-ChLC”) oligomer that was functionalized with azobenzene to generate different nematic LC alignments with the same color ink in a single continuous direct ink writing (DIW) process without changing printing parameters, yielding a photoisomerizable elastomer [145].

The creation of cell-laden constructs by 3D printing of a light-sensitive ink based on alginate and photothermal polydopamine (PDA) yielded 3D structures that were able to change their folded shape under NIR stimulation and preserve their new morphology for at least 14 days. This shape-changing response could also be tuned by laser power and irradiation time. The biocompatibility of the hydrogel-based ink, the NIR-sensitive structures and conservation of the deformed shape make this approach attractive to apply in 4D bioprinting of artificial tissues and organs [146].

#### 4.1.2. Temperature

Temperature is one of the most frequently used external stimuli in 4D printing to produce a shape deformation and geometric arrangement of responsive materials, as it can be simply regulated and applied in a non-invasive way. The most extensively used temperature-responsive materials in 4D-printing applications are SMPs and sensitive polymer solutions. Generally, SMPs are first printed and then shape-modified with temperature [147,148,149]. They can be permanently deformed applying a temperature above their glass transition temperature (Tg,1), or temporarily modified by heating at a temperature between Tg,1 and their intermediate glass transition temperature (Tg,2) and subsequently cooling to a lower temperature. The recovery of the original morphology occurs when the polymer is reheated at an elevated temperature. This ability to recover its form after the applied stimulus can be particularly useful for the repair and recovery of bone defects [150,151]. This approach only functions with polymers whose Tg is lower or equivalent to body temperature. For example, 3D-printed polylactide (PLA)/hydroxyapatite (HA) porous scaffolds that have the capacity to recover their shape after compression–heating–compression cycles for a potential use in self-fitting tissue engineering implants [152,153]. Poly(glycerol dodecanoate) acrylate (PGDA)-based SMPs with a transition temperature between 20 °C and 37 °C were developed to program the shape at room temperature and modify it at human body temperature [154]. These materials showed a shape-fixity ratio of 100% at 20 °C and a recovery ratio of 98% at 37 °C, demonstrating through in vitro and in vivo tests the excellent adaptability of the 3D-printed PGDA constructs for biomedical implantation.

Sensitive polymer (SP) solutions react to a temperature change by disrupting the hydrophilic and hydrophobic interactions between the SP chains and the solvent [155]. This results in the precipitation, shrinkage, or expansion of the SP. Some of the most common SPs used for 4D printing are poly(N-isopropylacrylamide) (PNIPAM), PEG, collagen, gelatin, and poly(N- vinyl caprolactam) (PNVCL). For example, PNIPAM-based hydrogels have a low critical solution temperature (LCST), so at T < LCST the hydrogel network expands due to the absorption of water, while at T > LCST the hydrogels eliminate the liquid and contracts. This temperature-dependent behavior has been exploited to develop 3D-printed mobile micromachines of PNIPAM that can penetrate and control obstructions of narrow channels [156]. They demonstrated that these microrollers and microscrews prepared by two-photon polymerization-based 3D printing can reversibly swell and shrink with temperature. Indeed, these size-controllable micromachines of PNIPAM can swell with temperature up to 65% of their initial length, making them attractive to target obstructive interventions. PEG has also been used for the development of 4D-printed shape memory implants. PEG1.5k was combined with PLA to obtain a biocomposite with a controllable transition temperature that can act as a biomimetic intestinal stent [157]. By fused deposition modeling, they printed functional 4D implants that remain in an elastic condition during the implantation process to decrease tissue damage and continue in a glassy state at body temperature (after implantation), providing appropriate support to the wound. These biocompatible intelligent stents have the ability to reconfigure at 37 °C and reopen the blocked colon. PEG was also combined with gelatin and a hyperbranched triethoxysilane reagent (HPASi) to 3D print a thermoresponsive material that shows good biocompatibility, improved mechanical strength, and the ability to fold and unfold [158]. Indeed, the material was forced into a temporary shape of “V” with a folding angle of 171°, and after heating it, the hydrogel immediately recovered its original unfolded shape after a few seconds.

#### 4.1.3. Electric Field

Electroactive biomaterials are exciting for a variety of biomedical applications including drug delivery [159], tissue engineering, and regenerative medicine [160]. In this sense, electrically conductive materials have also been investigated in 4D-printing paradigms [161]. The development of electroactive materials requires the inclusion of conductive components such as CNTs, carbon fibers, carbon black, graphene or gold, silver, nickel, copper nanoparticles, conducting polymers, etc. For example, the 4D printing of electro-responsive composites based on thermoplastic polyester urethane (PEU), PLA, and multiwall CNTs (MWCNTs) [162], wherein the MWCNTs act as fillers to make the polymeric material electroactive and induce Joule heating to trigger shape change after applying an electric current. Four different structures were printed with electro-active filaments and the triple-shape effect (TSE) was studied, demonstrating that after applying a low voltage to a U-shape composite containing 14% of MWCNTs, the temperature increased to 50 °C and the original form was partially recovered. However, when a higher voltage was used, increasing the temperature to 90 °C produced a near-complete recovery of the shape.

Printing composites of CNTs and PLA by the fused deposition method yielded 4D electroactive devices [163]. The form recovery behavior of conductive filaments and the effect of structural design on the shape memory electro-response of 4D-printed structures was studied. Filaments with a higher concentration of CNTs exhibited an enhanced electrical and thermal conductivity which resulted in an improved electroactive shape memory performance. The 2D and 3D structures printed with the most conductive filaments also showed excellent electroactive response with a 90% shape recovery under voltage application. This work shows the potential of 4D technology to develop new responsive devices with multiple applications.

Finally, 4D-printed electroactive nanocomposites with shape memory behavior were developed through direct ink writing for liquid sensors [164]. The combination of poly (D,L-lactide-co-trimethylene carbonate) (PLMC) with CNTs generated 4D-printed materials with a fast electro-responsive shape-changing performance and high electrical conductivity. They demonstrated that after applying a 25 V voltage, the temperature of the material increased to 80 °C in 47 s, producing the recovery of the scaffold’s original shape within 16 s. As the relative resistance change (RRC) of the material shifted when it was submerged in a solvent due to a variation in the connection between CNTs, they used the 4D-printed PLMC/CNTs-based scaffolds to sense different liquids. Furthermore, they improved the sensor, making it able to deform and adapt its shape to the liquid environment under an electric field application.

#### 4.1.4. Magnetic Field

The remote and safe control of the 4D-printed constructs can potentially also be achieved by an applied magnetic field from a certain distance [165]. Magnetism-responsive materials can be obtained by the incorporation of ferromagnetic or paramagnetic additives to the polymeric structure that can actuate under a magnetic field trigger. Some of the most common magnetic additives are metal alloys, metal oxides, and magnetic nanoparticles. By the addition of any of these magnetic-response components, the 4D-printed material can experience a shape change through direct magnetism, thermomagnetism [166], or electromagnetism [167]. To develop 4D-printed magnetoactive soft materials with a controllable shape change and movement capacity, an ink based on hard magnetic microparticles of NdFeB, polydimethylsiloxane (PDMS), dibutyl phthalate, and fumed silica was printed to yield 3D magnetoactive soft materials [59]. This material was then magnetized by a pulse strong magnetizing field Hm, and its shape could be controlled by applying an external magnetic field. Through this method, a bionic human hand, bionic butterfly, and bionic turtle were produced, which could move their fingers, wings, and legs, respectively, via on-demand magnetic activation.

A PDMS-based ink with Fe nanoparticles was printed to create 4D magnetoactive structures with a fast response to an external magnetic field [135]. Due to the mechanical flexible properties of the PDMS, the 3D structure obtained could deform and recover its original shape after applying or removing the magnetic stimuli. To demonstrate the fast reaction of the 4D-printed PDMS/Fe composite under a magnetic trigger, a butterfly structure was printed and the speed of movement of its wings under an external magnetic field provided by NdFeB magnets was studied, finding that the 3D butterfly wings could move up and down in only 0.7 s and the flapping speed increased with a faster movement of the magnets. The low magnetic coercive force and the high magnetic permittivity of the iron nanoparticles used for the ink could explain the capacity of the 4D-printed material to respond rapidly under an external magnetic field, which makes it promising for functional biomedical devices. Other recent reported works describe the development of 4D-printed magneto-responsive SMMs by combining thermoplastic PU (TPU) with PLA and Fe_3_O_4_ particles [168], or with NdFeB [169]. Both intelligent 4D-printed structures exhibited a high tensile strength and fast response under a remote external magnetic field. These findings lay the basis for future studies on magnetoactive structures from 4D printing.

### 4.2. Chemical Stimuli

#### 4.2.1. pH

pH variation is one of the most used chemical stimuli that can impart 3D-printed materials with interesting shape transformations. pH-responsive materials are widely employed for several biomedical applications, especially for those treatments where the material is exposed to different pH conditions, as happens in cancerous or inflammation sites. Upon pH variation, the 3D-printed materials can swell, shrink, dissociate, or collapse due to a change in the chemical interactions or bonds of the material. Both natural and synthetic polymers are used to develop pH-sensitive 3D-printed materials [170]. Natural pH-responsive biopolymers include hyaluronic acid, collagen, alginate, gelatin, keratin, and chitosan [171], while synthetic polymers are predominantly composed of acrylic acid, histidine, and L-glutamic acid monomers [172]. These materials are also classified into basic polymers (with an −NH_2_ group) and acidic polymers (containing −SO_3_H and −CO_2_H groups). In a recent work, pH-responsive tablets were developed by semi-solid extrusion 3D printing to improve the bioavailability of oral drugs. By combining different ratios of carboxymethyl chitosan, sodium alginate, and PEG diacrylate (PEGDA), they prepared new polypills with a site-targeted and sustained drug release controlled by the pH of the stomach and intestine [173]. In this case, the secondary crosslinking of the material with Ca^2+^ gave the tablets an active response at different pH values. A pH-sensitive 3D-printed formulation was developed for colon-specific protein release [174]. For this, they used the digital light processing (DLP) printing technique to co-print constructs based on the reverse thermo-responsive poly (ethylene oxide)/poly(propylene oxide)/poly(ethylene oxide) (PEO–PPO–PEO) triblocks and the pH-responsive acrylic acid to provide them pH sensitivity. They demonstrated that the 3D-printed structures exhibited fast and reversible swelling–deswelling behavior depending on the temperature and pH of the environment. Bovine serum albumin was used as a model drug to test the multi-responsive activity of the material, showing that at pH 2.0 (below acrylic acid’s pKa) and 10 °C, the 3D material released only 20 wt% of the encapsulated protein after one week, whereas at pH 7.4 (above acrylic acid’s pKa) and 37 °C, it released 65 wt%. Given the different pH values that can be found in the human body, this feature can be exploited to create new pH-responsive devices for biomedical applications.

#### 4.2.2. Humidity

As well as pH-responsive scaffolds, moisture-sensitive materials can change their shape, function, and dimension under a variation in humidity [175]. Humidity-responsive materials can swell up or shrink in the presence or absence of liquid, which makes them attractive for 4D printing [176]. Although these features are interesting for biomedical applications, the transition procedure between the swollen and the shrunken state should be carefully controlled to keep the integrity of printed materials. A pallet of cellulose-filled filaments with different stiffness and hygroresponsiveness were 3D printed to generate hygromorphic structures using the fused filament fabrication technique [177]. Due to the high hygroscopicity of cellulose, all prototypes showed fully reversible cyclic opening and closing under different relative humidity conditions. These properties bring innovative ideas for the development of smart materials that can react autonomously in response to relative humidity shifts. Moisture-responsive polymers have also been used to fabricate a seed-like soft robot by 4D printing [178]. The artificial seed was obtained by the fused deposition modeling of hygroscopic PCL combined with the coaxial electrospinning of hygroscopic fibers composed by PEO and cellulose nanocrystals. The obtained robot was able to mimic the biomechanics of *Pelargonium appendiculatum* (L.f.) wild seeds and explore and adapt its morphology to the soil by varying the environmental humidity. This kind of hygroscopic actuator could perform as a renewable energy solution for miniaturized robots.

### 4.3. Biological Stimuli

Bioresponsive materials use biological stimuli such as glucose levels, enzymes, proteins, or other biomacromolecules to exhibit bioactive functions [179]. Apart from chemical and physical stimuli, 4D-sprinting technology also aims to produce materials with sensitive behavior when exposed to biological stimuli.

#### 4.3.1. Enzymes

Enzymes are highly specific and selective molecules which are involved in different biological processes. Thus, the interest in using printed enzyme-responsive materials for biomedical applications has increased over the years since many enzymes are found in the body [180,181,182]. For example, the incorporation of enzymes to 3D-printed materials can be used in the field of tissue regeneration to customize devices that fit patients well or induce the degradation of implants for their natural removal. In a recent work, a thermal paste printing technique was used to obtain PCL composites containing lipases to develop a new biomaterial that can be placed and naturally degrade inside the body [183]. Due to the capability of lipases to degrade biodegradable plastics, it was shown that 3D-printed PCL-lipase composites were hydrolyzed in an aqueous environment at 37 °C within a reasonable period of time. To demonstrate this, they imbedded PLA objects (that do not react with lipases) inside the printed PCL-lipase structures. The hybrid composites were incubated in buffer at 37 °C and after 8 days they observed that the PCL-lipase shell was fully decomposed, releasing the embedded PLA objects intact. These kinds of 4D-printed materials may be applied as a biomedical implant where a fraction of the scaffold is required to be absorbed into the body before another part. To demonstrate the potential of enzyme-responsive materials in tissue engineering for bone reconstruction, alkaline phosphatase was included within 3D-printed PEGDA scaffolds to generate enzyme-induced in situ calcification [184]. The 3D-printed material containing the alkaline phosphatase was immersed in a solution of α-D-glucose-1-phosphate, resulting in the release of phosphate groups which, in the presence of free calcium, precipitate as calcium phosphate on the printed structures. Both microscopic analysis and colorimetric staining confirmed the formation of calcium precipitates. Thus, the strategy of immobilizing enzymes in 3D-printed structures is an attractive technique to control calcification processes, bringing an alternative method for bone restoration. Apart from tissue-engineering applications, printing immobilized enzymes also represents a new class of 3D-printed biocatalysts with a wide variety of uses in industrial biotechnology [185], like drug screening [186], organic synthesis [187], biosensing [188], and industrial and environmental applications [189,190].

#### 4.3.2. Biomolecules

Urea and glucose monitoring are nowadays a regular and simple procedure for early diagnoses or indications of kidney diseases and diabetes, respectively. Over the years, much effort has been made to develop simpler and more accurate biosensors to improve urea and glucose detection [191,192]. Among all the fabrication methods, 4D printing has emerged as an efficient technology to produce biomolecule-responsive materials for the quantitative analysis of urea and glucose in complex biological samples. In this way, a recent report used the digital light processing (DLP) 3D-printing method and photocurable resins with or without 2-carboxyethyl acrylate for the fabrication of an all-in-one needle panel meters for glucose and urea detection [193]. By combining the 4D-printed needle panel meters with the derivatization reactions for urea hydrolysis or glucose oxidization catalyzed by urease and glucose oxidase, respectively, the urea and glucose concentration can be quantified by measuring the bending of the needles. The advantages of this new device include the possibility of reuse, its applicability in complex biological matrices, its simplicity, cost-effectiveness, and its independence in relation to the analysis conditions (temperature, buffer, pH).

## 5. 4D-Bioprinting Applications

### 5.1. Advantages and Disadvantages of 4D Printing for Biomedical Applications

The use of 4D-printing technology has revolutionized the field of biomedical applications by providing novel solutions for different limitations of 3D printing (Table 1). The process of 4D printing enables the manufacturing of smart materials with stimuli-responsiveness and shape-changing behavior. These 4D-printed bio-architectures can change their properties as a function of environmental conditions or external stimuli and present dynamic and flexible structures—like living tissues—which is essential for biomedical applications such as tissue engineering and drug delivery. Furthermore, 4D-printing technology has other advantages such as the spatio-temporal control of printing procedures and the use of the intelligent stimuli-responsive materials to fabricate patient-specific products [194].

However, despite all these advantages, 4D-printing technology has some limitations that restrict its use in specific biomedical applications such as drug delivery, regenerative medicine, and implantable devices [195,196]. For example, materials do not always present the required strength, flexibility, and durability, or they are not able to resist the extreme conditions used during the printing processes. In addition, it is difficult to find materials with shape-changing behavior and which at the same time exhibit biocompatible properties and appropriate degradation rates that guarantee the protection of patients. Although 4D-printing technology has the ability to produce structures with different properties by combining diverse materials into one printed object, it is often difficult to find materials that can be combined with each other without affecting the desired functionality of the final device. Also, some current 4D-printing methodologies either do not have the resolution and accuracy required to fabricate materials that properly fit within the patient’s body or are expensive, time-consuming, and are not scalable, making them inappropriate for large-scale production to satisfy the needs of numerous patients. Finally, given the innovation of 4D-printing technology, the regulations and ethical considerations that ensure the safety and efficacy of the 4D-printed biomedical devices, protect patient privacy, and ensure the protection of delicate data are still not clear.

In this section, some of the most recent and innovative works that have been developed using 4D technology for biomedical applications are described.

### 5.2. Light-Responsive Materials

The advancements in 4D-printing technology have revolutionized the fabrication of 3D-printed structures by allowing shape and functional changes over time. This cutting-edge technology offers a myriad of practical applications in tissue engineering, cell encapsulation, wound healing, and drug delivery through the creation of complex and light-responsive multilayer scaffolds [197,198,199].

While the human body has the innate ability to regenerate damaged tissue, certain types of tissue, particularly critical-sized defects, pose significant challenges due to their complex design [200]. Bone tissue regeneration, for instance, is one of the most challenging issues to address because of its intricate architecture, including an interpenetrating vasculature network and calcified areas [201]. In this regard, 4D scaffolds have emerged as a promising solution, providing a dynamic and functional environment that can be tailored to meet patient-specific requirements such as pore size and tissue structure [202,203]. One of the key methods to achieve these demands is through the use of light-responsive polymeric chains to obtain photoisomerizable and photodegradable scaffolds [204]. A direct-ink-writing strategy using a photo-crosslinkable polymer of GelMA was used to create tissue scaffolds that support neoangiogenesis [205]. The construct is composed of a central rod printed with GelMA with low methacryloyl substitution and a high rate of degradation, forming the vessel channel. The walls were printed using highly functionalized GelMA-filled silicate nanoplatelets and various amounts of VEGF to promote osteogenesis and micro-capillary formation, forming a gradient. The bioink includes human mesenchymal stem cells (hMSCs), which differentiate into smooth muscle cells in the outer layer and inner fiber, supporting endothelial cell proliferation and osteogenic development, and promoting the formation, stability, and maturation of vascular vessels in vitro. Cells can be perfused through the inner channel, and a mature bone niche can form after 21 days.

**Figure 2:**
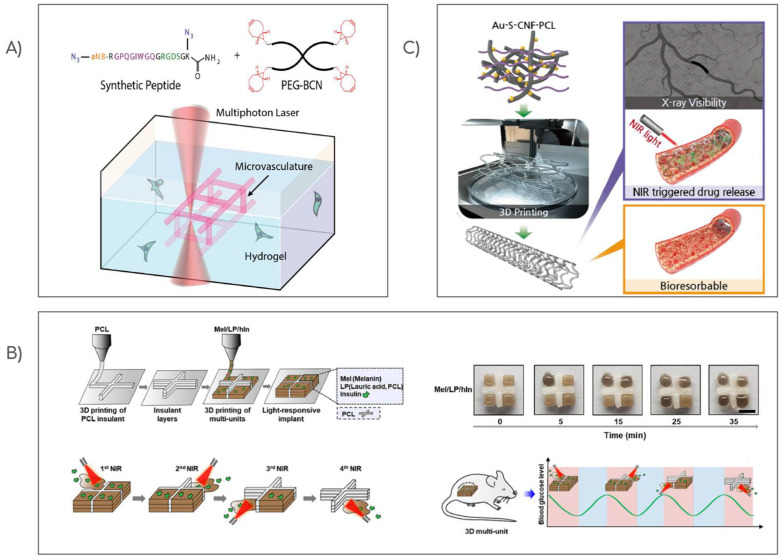
Schematic illustration of different light-responsive scaffolds for tissue engineering and drug delivery. (**A**) Microvasculature fabrication through multiphoton lithography within multipurpose hydrogel biomaterials elaborated with PEG tetra bicyclononyne (PEG-tetraBCN), and a diazide-modified synthetic peptide. Reproduced from [206] with permission from John Wiley and Sons. (**B**) NIR-triggered on-demand delivery of insulin (hIn) from 3D-printed multiunit implants (LP, lauric acid, and polycaprolactone) at various times to regulate blood sugar levels in diabetic mice. Adapted from Kim et al., [211] with permission from Elsevier. (**C**) 3D-printed CDECS of polycaprolactone (PCL) reinforced with gold nanoparticles-decorated carbon nanofibers for real-time X-ray imaging and targeted therapies. Adapted from Jeong et al., [215] with permission from Elsevier.

A tissue-engineering challenge is achieving the dynamic behavior of blood vessels to fulfill the needs of the tissues during various repairing and remodeling processes. A technique for creating complex and programmable 3D vascular networks inside biocompatible hydrogels using precise molecular photolysis was described [206]. Strain-promoted azide-alkyne cycloaddition (SPAAC) between a diazide-functionalized synthetic peptide, tetrabicyclononyne (PEG-tetraBCN), and PEG was used to create photodegradable hydrogels. The crosslinker contained a photodegradable moiety (ortho-nitrobenzyl ester, *o*-NB) and the polypeptide sequence GPQGIWGQ, which can be cleaved by various types of metalloproteinases (Figure 2A). The tri-amino acid sequence, arginine–glycine–aspartate (RGDS), was also added to the peptide to promote overall cell proliferation, adhesion, and spreading. Using multiphoton-lithography inside the bio-printed structure, the effective and spatial-specific channel building, endothelialization of artificial vessels, and cytocompatibility of photodegradation by irradiating close to encapsulated hS5 stromal cells within the hydrogel was demonstrated. These results highlight the vast potential of these polymer-peptide-based materials for engineering dynamic and customizable tissues. In a similar approach, photodegradable hydrogels composed of various kinds of macromers and *o*-NB groups that encapsulated hMSCs was described [207]. By choosing the functionalization grade from *o*-NB linkers, they could manipulate the rate constants of degradation obtaining a composite light-sensitive hydrogel with selective erosion and different delivery profiles. They not only tested the cytocompatibility of the construct but also the differential release of various cell populations. This approach could be useful in the regeneration of complex tissues that cannot be healed by the activity of a single cell type. In addition, while implants must be able to address tissue damage, they must also overcome issues such as ruptures and positioning challenges within the affected area. Red-light-responsive (625 nm) hydrogels based on the interaction between azobenzene (Azo) and cyclodextrins (CDs) were prepared which exhibited reversible adhesion and mechanical properties, as well as self-healing capacity [208]. The hydrogels were prepared via crosslinking between CDs and photoisomerized tetra-ortho-methoxy-substituted Azo (mAzo)-functionalized hyaluronic acid (HA) to generate hydrogels with weaker photoisomerization and stronger hydrogen bonding, which can prevent a complete sol-gel transition. To evaluate the cytocompatibility and multifunctional properties of the hydrogel, NIH 3T3 cells were cultured on the hydrogel surface, and the MTT assay showed a high degree of cellular viability of more than 90% after 3 days of culture. The self-healing response of the hydrogel was demonstrated by cutting it and then irradiating it with a red-light source, which resulted in complete fading of the notch within 3 h, as opposed to when the experiment was conducted in the dark. To rule out a photothermal effect, the same experiment was carried out at a fluctuating temperature, and no response was observed. The researchers hypothesize that dynamic noncovalent binding and dissociation between CD-HA and mAzo-HA promote supramolecular hydrogels to self-heal. Additionally, irradiation of the hydrogel surface induced a reversible change in viscosity, allowing for light-controlled adhesion. This behavior is likely due to competition between the partial photoisomerization of the mAzo groups, which induces a softening process, and the strong hydrogen bonds between the HA chains and the substrate, which are responsible for adhesion [209]. Overall, these findings suggest a feasible strategy for developing a dynamic hydrogel with potential benefits for tissue engineering, which can be remote-controlled in the context of injured sites.

Light-responsive materials are abundant in the field of drug administration and tissue engineering due to their high stimulus/response accuracy, both spatially and temporally, and the transparency of tissues to NIR light [210]. Scaffolds with rectangular divided regions allowed for the controlled release of insulin from the matrix upon NIR irradiation [211]. The partitions were made of polycaprolactone, while the responsive regions were printed using lauric acid, polycaprolactone, and melanin as the photothermal compound. In this case, local heating triggered insulin release from the matrix (Figure 2B). The scaffold was effectively implanted in a mouse diabetic model, and after NIR irradiation, the glucose blood levels decreased due to insulin-released activity, demonstrating the efficacy of the construct as an alternative to repeated insulin injections for patients. Hyaluronic acid hydrogels that combine chemo-photodynamic therapy with light-triggered release of the anticancer drug DOX and photosensitizer PpIX were prepared [212]. The hydrogels were designed to degrade in response to reactive oxygen species (ROS) generated by photodynamic therapy (PDT). The researchers chemically conjugated PpIX to a dihydrazide-modified HA hydrogel (HA-ADH) to enhance the photosensitizer’s solubility and create a PDT-compatible scaffold. They subsequently crosslinked the HA-ADH with a ROS-cleavable dialdehyde-functionalized thioketal (TK-CHO) to obtain a ROS-degradable material. When the hydrogel is exposed to light, ROS generation is induced by the photosensitizer, which breaks down the ROS-cleavable small molecule crosslinker, resulting in localized PDT and on-demand release of DOX for subsequent chemotherapy. The study provides a promising approach to achieve the effective, localized delivery of anti-cancer agents. Unlike the development of metallic stents and polymer-based drug-eluting cardiovascular scaffolds (DECS) with dual functionality, the worldwide prevalence of cardiovascular disease, particularly atherosclerosis, characterized by reduced blood flow and artery narrowing due to plaque formation on the arterial wall [213], has demanded the development of novel treatments. Recent studies have shown the elaboration of controlled drug-eluting cardiovascular scaffolds, which offer improved qualities over traditional DECS, including decreased toxicity, controlled dosage release, and superior biocompatibility [214]. For example, 3D-printed drug eluting cardiovascular scaffolds composed of polycaprolactone (PCL) impregnated with gold nanoparticles-decorated carbon nanofibers that improve the mechanical properties of PCL scaffolds and provide controlled drug release and X-ray visibility were produced (Figure 2C) [215]. The proof of concept was carried out using DOX as a model drug. The release of the therapeutic agent was evaluated both in vitro and in vivo by implanting the scaffold in the mouse thoracic cavity under NIR-light irradiation. The cardiovascular scaffolds proposed offer a platform for tissue engineering, atherosclerosis treatment, and other localized therapies, and have shown no cytotoxic effects in HUVECs and VSMCs adhered to the construct. Furthermore, in the pursuit of overcoming many of the limitations of traditional drug delivery methods, researchers have developed micro and nanorobots that can transport drugs to specific targets of interest [216]. These innovative systems have been designed to enable the directed movement of the drug carriers, which can be combined with controlled release mechanisms activated by external stimuli. With the ability to precisely target and release drugs at specific sites, these technologies hold great promise for enhancing the effectiveness of drug therapies [217]. To achieve these features, bio-printed chitosan microswimmers capable of being powered by a rotating magnetic field (10 mT) were reported [218]. These microswimmers also demonstrated triggered DOX release using an external light stimulus (3.4 × 10^−1^ W/cm^2^ and 365 nm wavelength). The microswimmers’ mobility in water was also demonstrated, and due to the chitosan matrix, no cytotoxic effects were observed.

Another important area where 4D bioprinting may provide solutions is the treatment of skin wounds, which are becoming a global emergency. Extrusion-based bioprinting with cell-loaded bioinks is the preferred method for wound care due to its cost-effectiveness, accessibility, and ability to replicate tissue complexity [219]. One of the most crucial goals in the area of wound dressings is the creation of a biocompatible, biodegradable antibacterial surface that may stop bacterial adherence, preventing the colonization of biofilms and subsequent infections [220]. Wool keratin (a biopolymer) was utilized to produce light-responsive 3D scaffolds with antimicrobial activity [221]. The leucine–aspartic–valine (LDV) and arginine–glycine–aspartic acid (RGD) adhesion sequences present in wool keratin make it a promising material for regenerative therapy. To enhance its antimicrobial properties, the researchers incorporated two distinct kinds of photosensitizers (Ps)—5,10,15,20-tetrakis[4-(2-N,N,N-trimethylethylthio)-2,3,5,6-tetrafluorophenyl]porphyrin tetraiodide (TTFAP) and Azure (AzA)—at varying concentrations within the pores of the keratin sponges. Upon exposure to a 500 W halogen–tungsten lamp (400 nm), ROS were produced, which inhibited the activity of microorganisms [222]. The researchers demonstrated the effectiveness of the responsive scaffold against both Gram-positive and Gram-negative bacteria, with TTFAP showing a greater antibacterial response. Importantly, the scaffold did not exhibit any cytotoxic effects on fibroblast cell cultures, highlighting its potential for wound healing and tissue-engineering applications. Additionally, current research in this area is focused on developing multifunctional wound dressings that combine expertise from the tissue engineering and drug delivery fields. Hydrogel core/shell-based scaffolds with the controlled release of doxorubicin for cancer therapy were reported [223]. The core is composed of alginate–gelatin hydrogels coated with polycaprolactone (PCL) and PDA to achieve an on-demand drug release mechanism through near-infrared (NIR) laser activation. The PDA coating’s antioxidant properties and hydrophilic surface make the core/shell structure highly promising for wound treatment. The scaffold’s effectiveness for wound treatment was assessed using a full thickness wound model in rats, scaffold implantation, and subsequent histology and immunohistochemistry of the tumoral environment.

An artificial light-responsive 3D-printed beating heart with precise spatiotemporal modulation has been successfully fabricated during the last year [224]. In this sense, a biomimetic aortic valve microstructure composed of a buckyball unit of cells and a solid layer was printed. The buckyball’s unit of cells with a shape-morphing response under the stimulation of a near-infrared light beam were assembled to a dynamic and static layer made of CNTs-doped N-isopropylacrylamide (NIPAM) composite hydrogel resulting in a light-driven intelligent micromachine with enhanced light absorption, thermal conductivity, and mechanical modulus. In the absence of light, the valve kept closed but after a laser beam stimulation, the entire valve became an open state due to the efficient light–temperature conversion and thermal conductivity of the smart hydrogel. Using this technology micropillar cilia, microheart valves, and microclaw grippers can also be manufactured for biomedical, soft robotics and bionic structures applications.

In conclusion, 4D printing is an exciting area of research that has the potential to revolutionize the field of additive manufacturing. One of the most promising developments in this area is the use of light-responsive materials, which can be programmed to change shape or properties in response to different wavelengths of light. This technology has many potential applications, including biomedical devices, robotics, and smart textiles. As researchers continue to refine the techniques and materials used in 4D printing, it is likely that we will see even more innovative and transformative applications in the future.

### 5.3. Thermo-Responsive Scaffolds

Tunable mechanical properties are of the upmost importance for in vivo tissue-engineering applications of 3D-bioprinted materials. Thermo-responsive bioinks offer an interesting range of possibilities in this regard. Many examples of applications of 3D-bioprinted thermo-responsive polymers are in the cartilage and bone repair fields. Microporous scaffolds based on SMPs prepared by the combination of polycaprolactone triol, poly(hexamethylene diisocyanate), and castor oil revealed to enhance the attachment, proliferation, and differentiation of human bone-marrow-derived mesenchymal stem cells [225]. These cytocompatible polymers displayed a controllable shape with a tunable glass transition temperature in a range from −8 °C to 35 °C. Their recovery speed was easily adjustable, with shape fixing at −18 °C or 0 °C and recovering their full shape at body temperature. In this case, printing was assisted by a sacrificial 3D mold, bypassing certain difficulties and requirements of direct 3D-printing techniques. Human bone marrow mesenchymal stem cells were also successfully encapsulated within 3D-bioprinted interpenetrating thermo-responsive polymer scaffolds based on PNIPAM and hydroxyethyl-chitosan, with the reinforcement of dithiol-modified graphene oxide nanosheets [226]. By modifying the polymer components’ weight ratio, LCST could be easily tuned allowing cell encapsulation around 20 °C. The hydrogel was then fully formed at body temperature. The incorporation of graphene oxide nanosheets contributed to improving the hydrogel mechanical properties (extrudability and uniformity), providing an appropriate microenvironment for cell growth, and favoring cell viability inside scaffolds. Cell-laden polymer solution injection into rat necks demonstrated in situ hydrogel formation, and angiogenic activity was evidenced 28 days after administration.

The fine-tuning of the thermo-responsive mechanical properties of triblock copolymers of PEG and partially methacrylated poly[N-(2-hydroxypropyl) methacrylamide mono/dilactate] incorporating polysaccharides, such as methacrylated chondroitin sulfate or methacrylated hyaluronic acid, allowed the preparation of stable 3D-bioprinted scaffolds for cartilage repair [227]. The thermo-responsive behavior of these kinds of copolymers allowed the easy incorporation of drugs or cells at low temperatures and good printability at physiological conditions. The incorporation of polysaccharides resulted in an improved thermosensitive profile, increased storage modulus, and decreased degradation. Particularly, the addition of methacrylated hyaluronic acid improved printability and cell viability. Abundant cartilage matrix formation (chondrogenesis) was evidenced in chondrocyte-laden 3D-printed constructs using the polymer combination. Chondrocyte-laden, stratified cartilage scaffolds were also 3D printed using a thermo-responsive bioink blend composed of PNIPAM-grafted hyaluronan and methacrylated hyaluronan [228]. Polymer gelation at body temperature was based on the LCST of PNIPAM and ensured good printing fidelity, while allowing cell and growth factor loading at room temperature. Meanwhile, methacrylated hyaluronan was demonstrated to reinforce the construct mechanical stability for extended periods after post-printing UV exposure. The gelation temperature, storage modulus, and swelling behavior were shown to be influenced by the addition of cells or by different biopolymers such as chondroitin sulfate methacrylate or hyaluronan methacrylate. Importantly, to ensure the good viability of the encapsulated chondrocytes, PNIPAM-grafted hyaluronan should have been eluted by a washing step at 4 °C to produce a more porous scaffold that ensured good nutrient diffusion.

**Figure 3:**
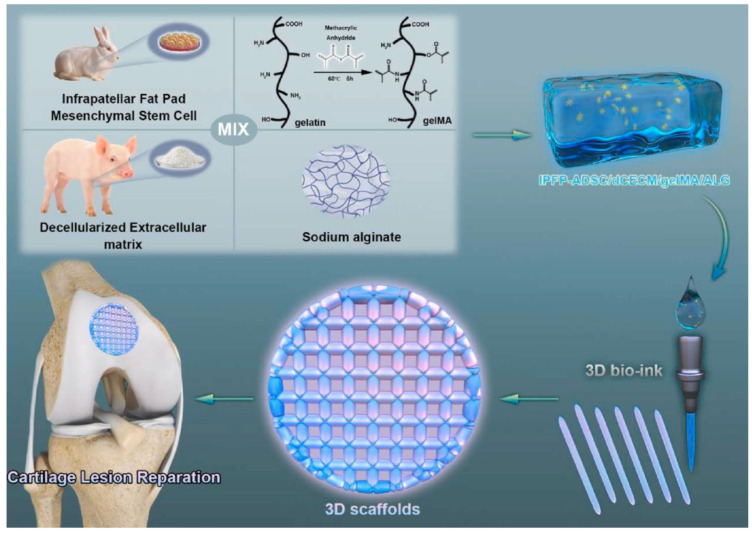
Scheme illustration of 3D-bioprinted scaffolds of decellularized cartilage extracellular matrix, methacrylated gelatin, sodium alginate, and infrapatellar fat pad mesenchymal stem cells, for articular cartilage lesion repair. Reproduced from [229] with permission from Elsevier.

More research on articular cartilage damage repair has recently been derived on the preparation of a temperature-responsive hydrogel bioink containing infrapatellar fat pads adipose-derived stem cells [229]. These cells were selected as they are supposed to have a similar chondrogenesis potential to that of stem cells from hyaline cartilage. The hydrogel bioink for 3D printing was composed of decellularized cartilage extracellular matrix, methacrylated gelatin, and sodium alginate. The thermo-responsive behavior, mechanical properties, degradation, and swelling behavior were influenced by the amount of decellularized cartilage extracellular matrix incorporated into the bioink. The optimal proportion was selected for the sake of achieving a low biodegradation rate, high mechanical properties, good printability, and histocompatibility. The printed scaffolds achieved an efficient and homogeneous integration of stem cells and bioactive factors within the generated network. Chondrogenic differentiation in vitro was demonstrated to be enhanced by the analysis of growth factors’ expression. When the scaffolds were subcutaneously implanted in an articular cartilage defect in vivo, they showed no immunogenic response and achieved full restoration of the damaged tissue, showing promise as a treatment for osteochondral lesions (Figure 3) [229].

Meanwhile, applications in bone repair include the development of a thermo-responsive polymer bioink based on poly(lactic-co-glycolic acid)-PEG [230] as a filler for bone defects. The resulting mechanical properties of constructs (yield stress and Young’s modulus) made it like cancellous bone. Moreover, this bioink allowed an easier incorporation of proteins and human mesenchymal stem cells at room temperature, while exposure to body temperature induced the formation of a rigid porous construct. In a more recent development for bone repair, a poly(organophosphazene) nanocomposite bioink with controlled mechanical properties in the range from 20 to 37 °C did not require post-crosslinking, and non-toxic biodegradation was achieved [231]. To stimulate bone regeneration, including cell migration and osteogenesis, the bioink was loaded with bone morphogenetic protein-2 (BMP-2) and transforming growth factor-beta1 (TGF-β1), which were retained by hydrophobic and ionic interactions into the porous structure. Simple mixing was needed to incorporate all bioink components below the gelation temperature. Above this temperature, the bioink mechanical properties (rheological properties and extrudability) were finely tuned, allowing the performance of the bioprinting process. Printing outcomes were also favored by the shear-thinning behavior and the self-healing ability of the prepared bioink. Moreover, after printing, no crosslinking was required owing to the optimal bioink behavior (mechanical stiffness) at body temperature and the consequent maintenance of the 3D structure for prolonged periods. When the printed scaffold was implanted in a calvarial bone-defect model, it was infiltrated by cells from surrounding tissues which promoted osteogenesis, healing the bone defects. After scaffold degradation over 6 weeks, which was followed by growth factors’ release, newly formed bone was observed.

**Figure 4:**
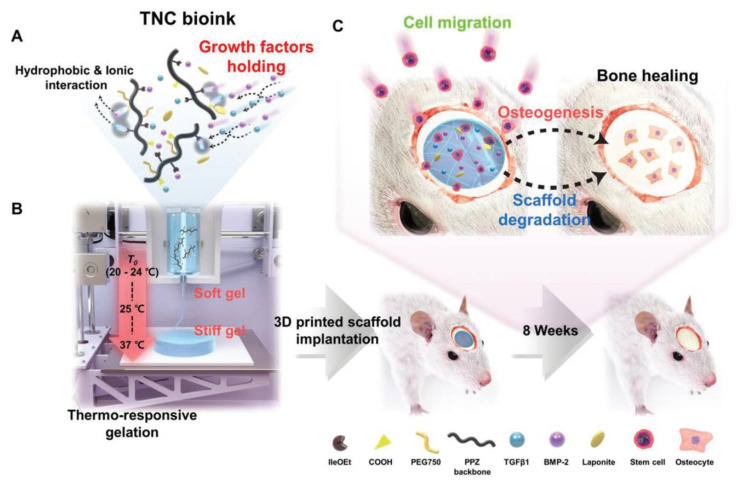
Schematic illustration of a thermo-responsive nanocomposite bioink system and bone tissue regeneration. (**A**) The bioink exhibits thermo-responsive behavior and incorporates growth factors through hydrophobic–ionic interactions. (**B**) After printing at 25 °C, the bioink reaches optimal properties at body temperature. (**C**) The bioink scaffold was implanted into a rat calvarial defect model, stimulating cell migration and osteogenesis for bone healing. Reproduced from Kim et al., [231] with permission from John Wiley and Sons.

Applications of 3D-bioprinted thermo-responsive polymers also extend to diverse areas, such as wound healing, neural tissue engineering, and cell spheroid formation. In this regard, polycaprolactone-PEG-polycaprolactone triblock polymers have proven to exhibit a good compatibility for endothelial cells and fibroblasts, which are commonly used in skin regeneration and wound repair (Figure 4) [232]. Composition ratio variation in copolymer blocks allowed the tuning of bioink mechanical properties and phase-transition behavior in the range from 10 to 40 °C. Polycaprolactone block crystallization has been shown to enhance bioink stability and mechanical strength, allowing printing by temperature control and the maintenance of structure afterwards. In another venue, PU nanoparticles with the inclusion of oligodiols [polycaprolactone diol, poly(L-lactide) diol and poly(D,L-lactide) diol] as soft segments determined a strong temperature-dependence response in the resulting hydrogel and served as appropriate 3D scaffolds for neural stem cells’ proliferation and differentiation. In a first study, these particles were mixed with soy protein to enhance the structural integrity and achieve rapid gelation and a good biocompatibility [233]. These biodegradable polymer hydrogels also exhibited a photo-thermal behavior owing to the presence of acrylate groups. In a further study, when PU dispersions were cured under UV treatment, they underwent gelation at body temperature with resulting moduli in the range of 0.5 to 2 kPa [234]. They showed shear thinning behavior, creep recovery, and dynamic viscoelasticity values at 37 °C that favored bioink 3D-printing processing. The printed constructs were soft but showed structural stability and printing fidelity. Neural stem-cell-laden scaffolds were easily prepared by mixing cells with bioink before printing. Hydrogels with a low modulus (less than 1 kPa) were more suitable for achieving neural stem cell survival, fast proliferation, and differentiation into neural cells as shown by the enhanced expression of specific neural-related genes.

The promotion of cell spheroid formation for drug screening or injectable formulations was also enhanced by responsive bioprinted materials based on sodium alginate grafted with PNIPAM-co-N-tert-butylacrylamide [235]. This copolymer was found to exhibit a bi-phasic gelation process. The first step involved the alginate chelation with calcium ions generating a soft gel network, while the second step involved the interaction of PNIPAM side chains upon heating, forming a more rigid structure. These phenomena resulted in an improvement in the storage modulus and benefited the 3D printability of the thermo-responsive bioink, the mechanical properties of which could be tuned by modifying the amount of calcium ions or the N-tert-butylacrylamide monomer. The printed scaffolds have been shown to promote the proliferation of human periosteum-derived cells in 3D and the formation of 3D spheroids. A facile spheroids recovery process ensuring their viability was possible owing to the thermally-induced reversible bioink behavior.

### 5.4. Electric-Field-Responsive Scaffolds

**Figure 5:**
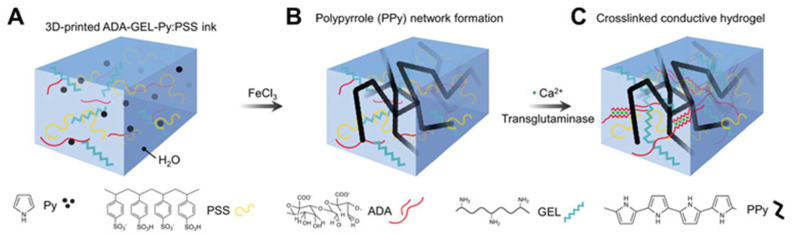
Schematic illustration of the formation of the ADA-GEL-PPy:PSS conductive hydrogel. (**A**) An ADA-GEL hydrogel precursor containing different molarities of Py and PSS is prepared. (**B**) After 3D printing, the formation of PPy is triggered by the oxidation of Py by immersion in FeCl_3_ solution. (**C**) The final ADA-GEL-PPy:PSS scaffolds are dually crosslinked using Ca^2+^ and microbial transglutaminase to respectively crosslink the oxidized alginate and gelatin network inside the hydrogel. Adapted from [239] with permission from John Wiley and Sons.

Electric-field-responsive 4D-printed scaffolds represent promising materials for medicine with opportunities for controlled drug release and cell stimulation [160,236]. A variety of conducting polymers (i.e., PPy, polyaniline, poly(3,4-ethylenedioxythiophene) (PEDOT) and/or conductive fillers (i.e., nanoparticles, graphene, MXenes) has been successfully applied in the development of electric-responsive scaffolds and wearable sensors [237,238]. Composite materials comprising polyester urethane, PLA, and MWCNTs as conductive fillers were printed employing the fused filament fabrication method. The composites displayed a stepwise, tunable shape change process that may be triggered remotely on demand [162]. Alternatively, hydrogel precursors offering 3D-printability were prepared with a combination of pyrrole with a high-gelatin-content oxidized alginate–gelatin (ADA-GEL). Subsequently, the electroactive PPy:poly(styrenesulfonate) (PPy:PSS) was synthesized inside the ADA-GEL matrix by the oxidation of pyrrole (Figure 5). The formation of an interpenetrating matrix increased the conductivity and stiffness of the hydrogels, maintaining the capacity to promote cell adhesion and proliferation [239].

Alternative ink formulations based on PEDOT:PSS were employed to print tissue scaffolds etc. Post-treatment by ethylene glycol in combination with thermal annealing provided printed materials with electrical resistance and stability. In addition, collagen coating improved the biocompatibility and adhesion of fibroblast and cardiomyocytes cells. These findings highlight the importance and opportunities of post-printing treatments [240]. In addition, the application of electrical stimulus can be used to enhance the release of drugs [241,242]. A 4D-printed hydrogel based on agarose/alginate–aniline tetramer with the capability of tailored electrically controlled dexamethasone release for neurodegenerative diseases’ treatment. There was a passive diffusion of dexamethasone from the hydrogel to the surrounding medium; however, the amount of released dexamethasone increases with the amplitude of the electrical voltage. Moreover, the hydrogels showed no cytotoxicity and promoted the adhesion and proliferation of PC12 cells, showing promise for neurodegenerative diseases’ treatments [243].

A recent work reported the development of a stable composite actuator with high potential as a biomedical device based on the combination of magnetorheological elastomer (MRE) composites with 4D-printed conductive shape memory polymers [244]. Briefly, silicone resins were filled with strontium ferrite magnetic particles and a thin conductive carbon black polylactic acid (CPLA) was 4D printed and fixed as a core inside the composite. The authors demonstrated the bi-directional feature of the actuator. By controlling the magnetic field, different shapes could be achieved and used as an attachment-like hook system. In addition, the 3D-printed electroactive structure was used to demonstrate the capacity of the composite actuator as a remotely operated hook using different input voltages. In this sense, the actuator within a structure can be operated externally and bent to reach a complex target. Consequently, the electroactive shape memory actuator may be applied in biomedical devices for a diversity of supportive treatments, including tracheal splints and shattered bones.

### 5.5. Magnetic-Field-Responsive Scaffolds

**Figure 6:**
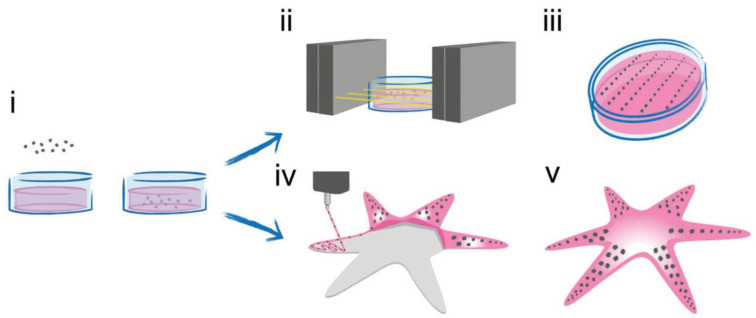
Schematic representation for the anisotropic nanocomposite fabrication and the 3D-printed star-shaped magnetic soft robot. (**i**) Iron oxide nanoparticles (IOPs) addition to a liquid suspension (T > 37 °C) of methacryloyl gelatin (G) hydrogel precursor. (**ii**) Application of a low-intensity magnetic field. (**iii**) Formation of the oriented IOPs filaments while the mixture temperature decreases below the melting temperature and final crosslinking via UV-light of the anisotropic nanocomposite. (**iv**) 3D printing of the G/IOPs mixture on a bed of G hydrogels. (**v**) The 3D-printed structure is finally crosslinked by UV light resulting in a stable star-shaped responsive structure. Reproduced from [246] with permission from John Wiley and Sons.

The 3D printing of structures that respond to magnetic fields has also been reported. The incorporation of magnetic nanoparticles to inks works as a nanofiller that alters their rheological and mechanical properties, but it also produces objects that can be remotely actuated via magnetic fields. Recently, an ink based on magnetic nanoparticles, alginate, and methylcellulose was employed to print 3D structures with mechanical stability and responsiveness to a magnetic field [245]. A low-intensity magnetic field was employed to align iron oxide nanoparticles into filaments within a gelatin methacryloyl matrix. Cells seeded on top or embedded within the hydrogel aligned themselves along the same axes of the aligned iron oxide nanoparticles’ filaments. Moreover, in the hydrogel, C2C12 skeletal myoblasts differentiated into myotubes without the addition of differentiation media. Interestingly, the nanocomposite hydrogel could be 3D printed to create complex heterogeneous structures that responded to magnetic fields (Figure 6). Indeed, the anisotropic distribution of the iron oxide nanoparticles allows the flapping movement of the soft gelatin-based hydrogel in response to alternated exposure to magnetic fields [246]. These results pave the way for the generation of bioinspired soft robotic systems.

**Figure 7:**
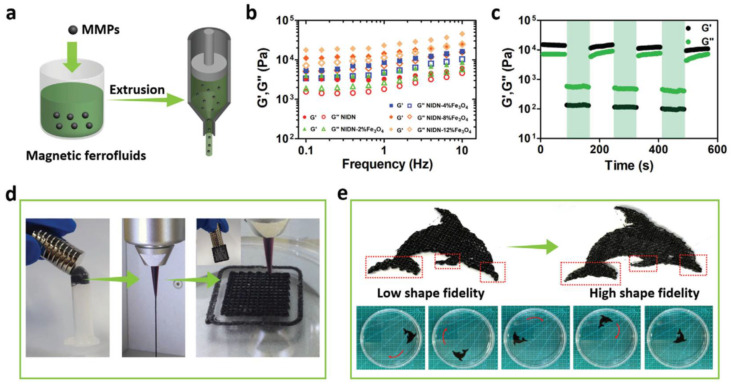
Preparation and characterization of magnetic nanoclay-incorporated double-network (M-NIDN) hydrogels for 3D printing and magnetic guided movement. (**a**) Schematic illustration of the preparation of M-NIDN hydrogel ferrofluids and the resulting extrusion filament. MMPs represent magnetic microparticles. (**b**) Oscillatory frequency sweep of the NIDN hydrogel precursors before and after the inclusion of magnetic microparticles. (**c**) G′ and G″ from continuous strain sweep with alternate high-strain (300%, shaded green) and low-strain (1%) conditions. (**d**) Digital images of the magnetic ferrofluids, extrusion filament, and 3D-printed lattice structure with magnetic-responsive behavior. (**e**) Top view of a 3D-printed magnetic dolphin robot with low or high filling density. The magnetic dolphin robot could be actuated by an outer programmed magnetic field (NdFeB magnet, N35). Reproduced from [247] with permission from John Wiley and Sons.

In a similar approach, Fe_3_O_4_ microparticles were incorporated in hydrogels precursors and it was observed that the increase in the content of microparticles provoked an elevation in the storage modulus (G′) and loss modulus (G″). This effect was beneficial to print a 3D structure with magnetic responsiveness. In this way, when the magnetic field was programmed to rotate, this induced the rotational movement of the magnetic dolphin robot (Figure 7) [247].

In another recent work, 3D-printed magneto-responsive shape memory polymers were fabricated by combining PLA, thermoplastic PU (TPU), and Fe_3_O_4_ particles [168]. The 3D-printed PLA/TPU/Fe_3_O_4_ showed a fast magnetic response, indicating the high efficacy of heat generation by magnetic particles, and a high recovery ratio. By designing and printing magnetic-responsive honeycomb and bionic flower-like models, the authors demonstrated the capacity of this novel material to be used in the development of multifunctional magneto-responsive printed devices with great potential as biomimetic structures.

### 5.6. Humidity-Responsive Applications

Water is another prevalent trigger. It has the potential to induce changes in bioprinted scaffolds through differential water sorption levels, thereby leading to swelling within distinct compartments of the scaffolds. This phenomenon causes bioprinted scaffolds to exhibit temporally- and spatially-dependent swelling patterns when submerged in water.

Hydrogel scaffolds composed of photocrosslinked PEG bilayers were developed where the PEG bilayers were composed of two distinct molecular weights and are crosslinked through conventional photolithography methods. The driving force behind this self-folding phenomenon is the differential swelling exhibited by the two PEG bilayers when immersed in aqueous solutions, resulting in the curvature of the scaffolds. Cells are then encapsulated within these PEG bilayers, and in the presence of water, the cell-laden scaffolds autonomously transform into cylinders of varying radii due to the water-induced self-folding of the PEG bilayers. The outcomes of these analyses indicate that the encapsulated cells maintain viability and robust insulin production for a period exceeding eight weeks within the cultured environment. Through meticulous design, such bioprinted 3D responsive scaffolds can be tailored to achieve complete customization, thus facilitating the construction of diverse microscale geometries that align with anatomical relevance [248].

The formation of droplet networks that function in aqueous environments yields novel structures termed “multisomes”, wherein the encapsulated droplets not only adhere to one another but also bind to the surface of the oil droplet, forming interface bilayers that facilitate intercommunication between droplets and their surroundings. This communication occurs through membrane pores. By altering the pH or temperature of the surrounding solution, the contents within the droplets can be released. The multicompartment nature of multisomes emulates tissue-like structures and offers significant potential in the domains of synthetic biology and medicine [249].

In a recent work, the hydrophobic nature of zein was exploited to control its self-assembly in the presence of water [250]. By the 3D printing of zein gel in a supporting bath of Carbopol containing different amounts of water in an ethanol/water mixture, the hydrophobic and hydrogen bonding of the plant protein could be modulated, obtaining materials with different functions. For example, when zein was printed in a supporting bath with a high concentration of water, a higher drug loading and faster drug release rate were obtained compared to the gels printed in Carbopol with a lower water content. As well as this, the degradation rate and porosity of the material were also controlled by changing the water concentration: zein gels 4D printed in supporting baths with a lower amount of water showed lower degradation rates. This modulation of the properties of the printed structures by controlling the hydrophobic self-assembly behavior is crucial for the development of biomedical materials for tissue engineering.

### 5.7. Enzymes Responsive Materials

In the context of 4D bioprinting, biological stimuli-responsive polymers tend to hold more prominence compared to materials that respond to physical or chemical triggers. This preference can be attributed to several factors. Firstly, biological signal molecules originate from cellular secretions or natural metabolic processes. Secondly, numerous diseases stem from the excessive expression of specific proteins at the molecular level or the anomalous metabolic patterns of biological signals. Hence, the identification of a specific polymer system capable of selectively responding to disease-associated biological signals holds the potential to yield exceptional nanomedicines. These substances could be designed to target pathological cells specifically, thereby addressing common medication side effects [251].

With the aim of creating a biomaterial that emulates the characteristics of vascularized alveolar bone, a 4D-printed PEGDA hydrogel was designed. In their study, the authors outline a novel strategy to produce 4D-printed objects possessing multiple biological functionalities. These functionalities are established by encapsulating two distinct enzymes, namely alkaline phosphatase, and thrombin, during the 3D-printing process. To achieve this, alkaline phosphatase and thrombin were blended with the PEGDA polymer precursor, subsequently becoming entrapped within the structure formed through printing. This unique approach leads to a 4D-printed object with the capacity to generate a range of bioactivities that hold relevance for in vitro tissue-engineering applications. Specifically, the entrapped alkaline phosphatase plays a role in enabling the localized and pre-programmed calcification of select parts of the 3D object. In addition, the diffusion of thrombin from the object initiates the formation of a fibrin biofilm, including living cells, directly on the surface of the 3D object. They observed that these enzymes, over time, may promote both the processes of calcification and fiber formation. The study thus demonstrated the potential of utilizing enzymes to drive the formation of calcified structures and fiber patterns, akin to natural blood vessels, in the 4D-printed hydrogel construct. This study stands as a compelling demonstration of the efficacy of 4D-printed hydrogels in realizing intricate 3D shapes that house multiple active enzymes. Through the utilization of alkaline phosphatase and thrombin in the context of 4D printing, the researchers have achieved a significant goal: the creation of a bioinspired object with multifaceted activities. In essence, this study not only underscores the potential of 4D printing for creating advanced bioactive constructs but also introduces a paradigm shift in the methodologies used for developing complex tissue-engineered constructs [252].

In another novel innovation, for the on-site determination of urea and glucose, a 4D-printed all-in-one needle panel meter was recently fabricated [193]. By adding 2-carboxyethyl acrylate to photocurable resins and coupling with a derivatization reaction such as the hydrolysis of urea (catalyzed by urease to decrease [H^+^]) or oxidation of glucose (mediated by glucose oxidase to increment [H^+^]), the [H^+^]-sensitive layer of the 4D-printed needle allows the quantification of urea or glucose by measuring the bending of the needles with a Vernier caliper or pre-calibrated concentration scales and simple observation. These 4D-printed devices can be used several times and can detect 4.9 and 7.0 μM of urea and glucose, respectively. This design has been shown to be able to quantitatively analyze glucose and urea in human urine, fetal bovine serum, and rat plasma samples.

**Table 1:** Applications of 3D-printed stimuli-responsive materials.

Stimuli	Material	Applications	Ref.
Light	GelMA-filled silicate nanoplatelets containing hMSCs	Promotion of the formation, stability, and maturation of vascular vessels in vitro	[174]
	PEG tetrabicyclononyne (Mn ~20,000 Da), diazide-functionalized synthetic peptide	Generation of endothelialized 3D vascular networks	[206]
	Hyaluronic acid functionalized with photoisomerized tetra-ortho-methoxy-substituted Azo crosslinked to cyclodextrins	Regeneration of functional multi-tissue complex under an external control	[208]
	PCL, lauric acid, and melanin	Controlled release of insulin	[211]
	Dihydrazide-modified HA hydrogel	Effective, localized delivery of anti-cancer agents	[212]
	PCL impregnated with gold nanoparticles-decorated carbon nanofibers	Controlled drug release	[215]
	Chitosan microswimmers	Controlled drug release	[218]
	Wool keratin modified with TTFAP and AzA	Wound healing and tissue-engineering applications.Antimicrobial activity	[221]
	Alginate-gelatin hydrogels coated with PCL and PDA	Controlled release of doxorubicin for cancer therapy	[223]
	CNTs-doped N-isopropylacrylamide (NIPAM) composite hydrogel	Biomimetic aortic valve microstructure	[224]
Temperature	PCL triol, poly(hexamethylene diisocyanate), and castor oil	Enhancement of the attachment, proliferation, and differentiation of human bone-marrow-derived mesenchymal stem cells	[225]
	PNIPAM, hydroxyethyl-chitosan, and dithiol-modified graphene oxide nanosheets	Angiogenic activity	[226]
	PEG and partially methacrylated poly[N-(2-hydroxypropyl) methacrylamide mono/dilactate] incorporating polysaccharides	Cartilage repair	[227]
	PNIPAM grafted hyaluronan and methacrylated hyaluronan	Chondrogenesis	[228]
	Decellularized cartilage extracellular matrix, methacrylated gelatin, and sodium alginate	Chondrogenesis	[229]
	Poly(lactic-co-glycolic acid)-PEG	Bone repair	[230]
	Poly(organophosphazene), BMP-2, and TGF-β1	Bone regeneration	[231]
	PCL-PEG-PCL triblock polymers	Skin regeneration and wound repair	[232]
	PU nanoparticles with the inclusion of oligodiols [PCL diol, poly(L-lactide) diol and poly(D,L-lactide) diol]	Promotion of neural stem cells’ proliferation and differentiation	[233]
	PU	Promotion of neural stem cells’ proliferation and differentiation	[234]
	Sodium alginate grafted with PNIPAM-co-N-tert-butylacrylamide	Promotion of cell spheroid formation	[235]
Electric field	Alginate–gelatin containing PPy:PSS	Promotion of cell adhesion and proliferation	[239]
	PEDOT coated with collagen	Electrical controlled drug release	[240]
	Agarose/alginate–aniline tetramer	Controlled dexamethasone release for neurodegenerative diseases’ treatment	[243]
	Magnetorheological elastomer composites with conductive carbon black polylactic acid	Supportive treatments, including tracheal splints and shattered bones	[244]
Magnetic field	PLA/TPU/Fe_3_O_4_	Development of biomimetic structures	[168]
	Gelatin methacryloyl with iron oxide nanoparticles	Generation of bioinspired soft robotic systems	[213]
	Nanoclay-incorporated double-network hydrogel with magnetite nanoparticles	Magnetic guided movement	[214]
Humidity	PEG bilayers	3D biological studies and tissue engineering	[248]
	Multisomes	Domain of synthetic biology and medicine	[249]
	Zein gel in a supporting bath of Carbopol	Development of controlled self-assembly biomedical materials	[250]
Enzymes	PEGDA hydrogel with alkaline phosphatase and thrombin	Formation of calcified structures and fiber patterns	[252]
	2-carboxyethyl acrylate added to photocurable resins and derivatized with urease and glucose oxidase	On-site determination of urea and glucose	[193]

## 6. Current Challenges and Future Prospects

Although there have been significant advancements in 4D bioprinting for tissue engineering and regenerative medicine applications (Table 1), many challenges remain for the widespread clinical implementation of this technology. Critical obstacles may appear when considering material properties. Designing bioinks with suitable viscosity and the ability to withstand the conditions of the printing process while maintaining the stimuli-responsive behavior after fabrication poses important difficulties. Moreover, the mimicking of the biochemical and mechanical properties for recreating microenvironments of native tissues, the incorporation of multiple cell types in the same construct, and the design of bio-printed materials that respond to different type of stimuli to emulate the in vivo situation are still difficult challenges for 4D-bioprinting technologies. The desired degradation behavior of 4D-bioprinted constructs could also be complex to achieve as it could be influenced by diverse intrinsic properties (composition, structure, processing conditions) and extrinsic factors (microenvironment, healing rate, and immune response). Current printing technologies are limited at this time in creating structures incorporating multiple materials with different properties and stimuli responsiveness. They are also limited in meeting the high levels of resolution and accuracy needed for some bioprinted constructs.

In terms of biocompatibility, from a general perspective, an effective clinical translation of 4D-bioprinted devices should require the use of non-immunogenic and non-toxic materials that guarantee patient safety. It is also of the upmost importance that the cells encapsulated within the structures remain viable after being exposed to shear stresses during the bioprinting process. For instance, the incorporation of nanoclay particles or the modification with bioactive peptides have been shown to improve cell viability within 4D-bioprinted materials [253,254].

Other significant challenges to be met up by 4D printing for regenerative medicine and tissue-engineering applications are scalability and mass production. For these purposes, printing processes should achieve time efficiency and cost-effectiveness for the definitive adoption and clinical translation of 4D-bioprinted constructs. In this sense, integration with additive manufacturing or injection molding are envisioned. Clinical applications of 4D-printed biomedical devices also require complying with the approval of regulatory agencies (e.g., FDA), where new guidelines could be needed to address the unique properties of these devices. In this complex scenario, preclinical studies, clinical trials, and post-market surveillance will be on track. Moreover, several ethical considerations including informed consent, patient privacy, and equitable access will arise for the clinical implementation of 4D-bioprinting technology.

Future directions in the realm of 4D bioprinting should present innovative solutions to urgent clinical challenges and pave the way for new avenues of research. Currently, a notable limitation lies in the sensitivity of most available polymeric materials and hydrogels, which respond to a single stimulus, or in the adaptability of those sensitive to multiple stimuli, which undergo simple deformations, such as folding, curling, and bending, creating difficulties for the use of these materials in the complex human environment. A pivotal advancement comes in the form of multi-material and multi-process printing, enabling the fabrication of intricate, multifunctional structures made up of specific regions with different thermal, electrical, or mechanical properties, capable of responding to diverse stimuli in different ways. In that sense, the integration of nanomaterials into shape-memory polymers and hydrogels shows promise for constructing multifunctional bioengineered tissues with exceptional biomechanical properties [17]. This progress has left an indelible mark on 4D-printing applications in biomedical engineering, smart materials, and soft robotics. Overcoming existing challenges, including the advancement of printing systems and ensuring material compatibility, will undoubtedly expand the potential of 4D-printing technology [255,256].

Environmentally friendly and biodegradable materials for 4D printing in biomedical engineering are becoming more and more popular as worries about the effects of materials and waste management on the environment grow. In order to create biodegradable synthetic polymers for 4D printing, researchers are actively investigating the utilization of naturally produced materials like chitosan or cellulose [257]. The adoption of these materials might lead to more sustainable healthcare practices and reduce the negative environmental effects of biomedical equipment.

In the context of organ design and tissue regeneration, it is imperative not only to enhance biocompatibility but also to establish interlinkages within vascular tissues. The vascular system of organs and tissues hinges on the interconnectedness between vascular cells and tissue-specific cells. This interconnection is contingent on cell types and contents, necessitating meticulous attention when designing co-cultured tissue regenerative systems. Despite strides in 4D-bioprinting technology, efforts are indispensable to enhance the biomechanical performance of vascular tissue grafts and replicate the biological functionality and structural complexity of in vivo vascularization. Moreover, the integration of vascular tissues into living material poses another challenge that warrants scientific attention. The fusion of living cells with 4D-printed structures holds promise for developing biohybrid systems that exhibit the desired characteristics of both biological tissues and engineered materials, potentially diminishing the need for organ transplantation.

In the realm of personalized medicine, 4D printing assumes a significant role by facilitating the fabrication of patient-specific devices and structures tailored to individual needs. This encompasses the development of customizable implants and prosthetics that precisely conform to a patient’s anatomy, as well as patient-specific drug delivery systems for the optimal dosing and targeted delivery of therapeutic agents. Moreover, the integration of cells directly derived from patients would efficiently prevent rejection from the immune system when bioprinted constructs were implanted in vivo. More efficient therapies and better patient outcomes might result from the broad use of 4D printing in customized medicine.

Furthermore, the incorporation of self-healing properties into 4D-printed devices could enable the structures to repair themselves in response to damage, thereby reducing the need for replacement or invasive surgeries [258]. Moreover, the improvement of these implants with the integration of sensors and electronics into these printed structures represents a promising area of research. More individualized and successful treatment regimens might be made possible by these electronic components, which could also follow the healing process, monitor, identify potential issues, and give feedback to healthcare personnel.

Future innovations are also centered on the synthesis, design, and application of materials and devices at the nanoscale, with the aim of reducing side effects, improving therapeutic efficacy, and enabling personalized medicine. The confluence of nanomedicine with 4D-printing technology promises to revolutionize healthcare and improve patient outcomes through novel approaches to illness detection, management, and prevention. 

For everything mentioned above, improvements in the current techniques are required. Advances in 4D-printing techniques, including the infusion of machine learning and AI-driven design, are paramount for predicting material behavior and guiding the selection of geometries, materials, and fabrication parameters to achieve desired functionalities. The judicious application of these technologies will undoubtedly lead to more streamlined and effective fabrication processes, resulting in the enhanced capabilities of 4D-printing technology across a spectrum of applications and industries [259,260].

The considerations discussed herein are essential for establishing 4D bioprinting as a groundbreaking technology in bioengineering. Nevertheless, the transition of these concepts from laboratory settings to real-world applications necessitates the optimization of both automation and scalability in the processes outlined above. Advancements in automated quality control systems, high-throughput manufacturing techniques, and the creation of standardized procedures and guidelines will accelerate the large-scale manufacture, increased affordability, and effective clinical translation of 4D-printed structures. Advances in scalable and automated manufacturing processes will surely be revealed by ongoing research, which will be crucial in realizing the full potential of 4D printing and enabling its smooth integration into a variety of applications.

## 7. Conclusions

The process of 4D printing is a rapidly developing technology that is revolutionizing the biomedical field. By employing stimuli-responsive materials 4D printing allows for the creation of objects that can change shape or function over time in response to environmental stimuli. The ability of 4D-printed constructs to adapt and respond to their environment holds immense promise for various biomedical applications, including drug delivery systems, biosensors, tissue engineering, and medical devices. Indeed, applications drive the appropriate selection of biomaterials and stimuli to achieve optimal efficacy for 4D-bioprinted scaffolds. This could be used to improve the efficacy and reduce the side effects of drugs, repair damaged tissues, or to create new organs that are not possible with traditional manufacturing methods. The potential applications of 4D printing in the biomedical field are vast and still being explored. As the technology continues to develop, it is likely that 4D printing will have a major impact on different medical procedures [261]. In conclusion, this groundbreaking technology opens up a plethora of possibilities for developing innovative biomedical solutions that transcend the limitations of conventional therapies.
